# Enhancing the Ductility and Properties of Non-Vulcanized Polylactic Acid-Based Thermoplastic Natural Rubber Using Acetyl Tributyl Citrate

**DOI:** 10.3390/polym17050601

**Published:** 2025-02-24

**Authors:** Donlaporn Koedthip, Ekwipoo Kalkornsurapranee, Karnda Sengloyluan, Ponusa Songtipya, Ladawan Songtipya

**Affiliations:** 1 Program of Food Packaging Technology, Faculty of Agro-Industry, Prince of Songkla University, Hat-Yai 90110, Songkhla, Thailand; donlaporn.koedthip@gmail.com (D.K.); ponusa.j@psu.ac.th (P.S.); 2 Division of Physical Sciences, Faculty of Science, Prince of Songkla University, Hat-Yai 90110, Songkhla, Thailand; ekwipoo.k@psu.ac.th; 3 Program of Rubber Engineering and Technology, Faculty of Engineering, Prince of Songkla University, Hat-Yai 90110, Songkhla, Thailand; karnda.s@psu.ac.th; 4 Center of Excellence in Bio-Based Materials and Packaging Innovation, Prince of Songkla University, Hat-Yai 90110, Songkhla, Thailand

**Keywords:** polylactic acid, natural rubber, acetyl tributyl citrate, mastication time, weight ratio, plasticizer

## Abstract

This study examines the effects of mastication time and the addition of a plasticizer (acetyl tributyl citrate (ATBC)) on the properties of non-vulcanized polylactic acid/natural rubber (PLA/NR) blends using a factorial design, along with the impact of changing the weight ratio of the blends. The results reveal the formation of plasticized PLA (P-PLA)-based thermoplastics with enhanced ductility. ATBC functions as both a PLA plasticizer and a compatibilizer in the binary PLA/NR system. However, improving compatibility requires the exclusive use of masticated NR with an appropriate mastication time (60 min) before blending. Optimal properties are achieved at a P-PLA/NR weight ratio of 90/10, maximizing the impact strength (~35.40 J/m) and toughness (~7.21 × 10^6^ MJ/m^3^). However, higher NR contents lead to reduced mechanical performance due to poor interfacial bonding. Thermal analysis reveals superior miscibility and dispersion in blends with a lower NR content (10 wt%), while the addition of plasticizers and NR leads to a decrease in the glass transition temperature (T_g_) of the blends. The results suggest potential applications for developing biodegradable products with enhanced flexibility and improved low-temperature performance. The incorporation of ATBC can enhance material properties without relying on conventional synthetic compatibilizers.

## 1. Introduction

Environmental impacts are currently a major concern across various industries, with plastic waste contributing significantly to pollution. Bio-based polymers offer a promising alternative to petroleum-based plastics in a range of industrial applications. Among these, the biodegradable polymer polylactic acid (PLA) is increasingly being utilized due to its eco-friendliness, nontoxicity, high mechanical strength, and structural versatility [1,2,3]. Additionally, the United States Food and Drug Administration (FDA) has approved PLA for use in food contact materials, demonstrating its safety and suitability for diverse applications [4].

Despite its advantages, PLA can become increasingly brittle over time, particularly upon exposure to stress or low temperatures. This, in turn, may lead to cracking or failure, limiting its suitability for structural applications that demand high flexibility and impact resistance. Alternative materials such as thermoplastic elastomers (TPEs), notably thermoplastic natural rubber (TPNR), have been developed to address this issue. The incorporation of natural rubber (NR) enhances the ductility of PLA by improving its ability to absorb energy [5]. Si et al. (2018) [6] found that PLA/vulcanized NR outperforms PLA/NR in terms of the elongation at break, toughness, and impact strength. Moreover, Chanthot et al. (2021) [7] noted that PLA/sulfur-cured vulcanized NR has a higher impact strength than PLA/unvulcanized NR. This improvement was attributed to vulcanization, which generates a large number of free radicals, enhancing the interfacial reaction between PLA and NR.

While the mechanical properties of vulcanized PLA/NR blends are higher than those of simple blends, vulcanized materials do not exhibit biodegradability [8]. In environmentally conscious applications, TPNR, produced via a simple blending process, is considered a viable alternative. Bitinis et al. (2011) [9] found that incorporating NR into PLA significantly improves its mechanical properties, involving the transition from brittle to ductile fracture in the material. This enhancement was attributed to NR particles acting as stress concentrators, with an NR content of 10 wt% yielding the highest elongation at break. Pongtanayut et al. (2013) [10] corroborated this finding, noting the maximum elongation at break (~24%) using 10 wt% NR. Additionally, Pattamaprom et al. (2016) [11] demonstrated that the impact strength of the PLA/NR blends peaks (~23.4 J/m) at the same NR content. Furthermore, by varying the weight ratio of PLA/masticated NR films (with NR contents ranging from 0 to 25 wt%), Chanthot et al. (2022) [12] found that the addition of NR can improve the toughness of PLA; an NR content of 15 wt% yielded the highest toughness due to good dispersion and an optimized NR domain size in PLA.

Although a small amount of NR can enhance the flexibility and impact resistance of PLA, overall, the resulting blend remains relatively rigid and difficult to bend due to the crystalline nature of PLA. This limitation restricts the use of PLA in certain products. To expand the potential applications of PLA-derived TPNR, researchers have proposed improving the material flexibility by plasticizing the PLA, particularly through the incorporation of epoxidized soybean oil (ESO). Burkov et al. (2021) [13] reported that adding ESO (0 to 15 php) to the PLA/NR blend significantly increases strain and reduces the NR phase size. However, based on a review of the literature, ESO does not represent an ideal plasticizer for enhancing the flexibility of PLA in comparison to acetyl tributyl citrate (ATBC). ESO can improve the elongation at break by approximately 68% upon the addition of 15 wt% ESO [11]; in contrast, using 10–25 wt% ATBC can increase the elongation at break by approximately 85–254% [14] due to the similar solubility parameter values of ATBC and PLA [15]. ATBC also demonstrates a superior ability to enhance the flexibility and toughness of PLA compared to other plasticizers, such as polyethylene glycol (PEG) [15,16,17,18,19] and triglycerides [20]. Moreover, ATBC is widely recognized as a non-toxic and eco-friendly plasticizer [15,21], further advancing green material technology by encouraging the use of sustainable and biodegradable polymers. It is widely used in food packaging, medical materials, cosmetics, and the toy industry [22] and is approved by European regulations for use in food contact materials [15]. Therefore, in this study, ATBC was used as a plasticizer in PLA/NR blends to elucidate its potential to enhance material performance, a previously unexplored topic with regard to PLA/NR blend systems. This makes it an intriguing area of study, as it provides valuable insights into the optimal conditions for preparing such materials, as well as the role of ATBC in the polymer blend system. Understanding how ATBC interacts within the PLA/NR matrix could lead to better control over the mechanical properties and overall performance of the material, paving the way for more efficient and sustainable applications.

Nevertheless, blending PLA with NR poses challenges due to the significant polarity difference between the two materials. PLA is a polar polymer with ester groups, while NR is a nonpolar hydrocarbon; this polarity mismatch results in poor interfacial adhesion and phase separation, leading to blends with inferior mechanical properties and reduced compatibility [23]. Additionally, the molecular weights of PLA (ranging from 50,000 to 550,000 g/mol) [24,25] and NR (ranging from 50,000 to 3,000,000 g/mol or higher) [26] differ significantly, further affecting their properties and compatibility in blends. Polymers of similar molecular weight generally exhibit better miscibility and interfacial adhesion due to improved chain interactions, resulting in more uniform and stable blends [5,11,27]. They also affect the rheological properties of the material, such as viscosity and flow behavior, which influence the uniformity of the blend and its ability to form a stable matrix [11]. Therefore, significant molecular weight differences can lead to phase separation and poorer mechanical properties. Previous studies have noted the importance of selecting polymers with compatible molecular weights to achieve desired blend characteristics. For example, Utara and Boochathum (2011) [27] improved the compatibility and mechanical properties of linear low-density polyethylene (LLDPE)/NR blends by reducing the molecular weight of NR through ozonolysis. Blending LLDPE with NR of a lower molecular weight (8.30 × 10^5^ g/mol) significantly improved compatibility compared to NR of a higher molecular weight (14.5 × 10^5^ g/mol), yielding the highest impact strength and elongation at break. Similarly, Zhang et al. (2013) [5] and Pattamaprom et al. (2016) [16] studied the effect of mastication time (0–15 min and 0–10 min, respectively) on the mechanical properties of PLA/NR blends, finding that an increased mastication time improves the elongation at break, toughness, and impact strength.

This research primarily aimed to investigate the optimal molecular weight (M_w_) of NR for fabricating TPNR using both unplasticized and plasticized PLA (P-PLA). The mastication time of NR was varied (0, 30, 60, and 90 min) to examine its influence on the material properties, and the M_w_ of the raw materials was analyzed using gel permeation chromatography. Additionally, this study explored the effect of the weight ratio of PLA or P-PLA to NR on the physical, morphological, mechanical, and thermal properties of the prepared materials. The experimental results were compared with those of neat PLA and P-PLA for a comprehensive analysis, facilitating an informed material selection.

## 2. Materials and Methods

### 2.1. Materials

PLA (Ingeo Biopolymer 4043D, NatureWorks LLC, Blair, NE, USA) was used in this study. The PLA material had a density of approximately 1.24 g/cm³, a melt flow rate (MFR) of 6–9 g/10 min at 210 °C with a 2.16 kg load, and a melting temperature (T_m_) in the range of 145 °C to 160 °C. Standard Thai Rubber (STR5L) was supplied by Chalong Latex Co., Ltd., Songkhla, Thailand, and ATBC (≥99% ester content) was purchased from Chanjao Longevity Co., Ltd., Bangkok, Thailand.

### 2.2. Preparation of Plasticized PLA (P-PLA)

To identify the optimal ATBC content, we investigated the effects of varying the ATBC concentration (from 0 to 15 wt%) on the mechanical properties of PLA. Incorporating 15 wt% ATBC significantly enhances the elongation at break and the toughness of PLA, as shown in Table 1. However, increasing the ATBC content beyond this level leads to plasticizer exudation on the surface. Consequently, 15 wt% ATBC was selected for the preparation of P-PLA before mixing with NR. PLA was first mixed with ATBC for 5 min at 160 °C using an internal mixer (HAAKE™ PolyLab™ OS system, Karlsruhe, Germany) with a roller rotor at a speed of 25 rpm, followed by mixing for an additional 5 min at a rotor speed of 55 rpm to homogenize the P-PLA. Sheet samples were then prepared using a compression molding machine (PR2D-W300L350 PM-WCL-HMI, Chareontut Co., Ltd., Bangsaothong, Thailand) at 2000 psi and 160 °C for 8 min. The temperature of the mold was subsequently reduced using water as a coolant for 10 min, followed by air cooling for 5 min.

### 2.3. NR Mastication

NR was masticated for various periods (0, 30, 60, and 90 min) using a two-roll mill at room temperature and a nip gap of ~0.3 mm. The masticated NR was then stored for no more than 1 day before mixing with PLA.

### 2.4. Preparation of TPNR

#### 2.4.1. Effect of ATBC and NR Mastication Time

The experiment was designed as a 2 × 4 factorial design to investigate the main effect of ATBC contents (0, and 15 wt%) and NR mastication time (0, 30, 60, and 90 min) using a PLA/NR ratio of 60/40 on the properties of TPNR. This specific formulation was chosen because it represents the worst-case scenario, given its higher rubber content and phase separation. The total number of experimental conditions is shown in Table 2. Initially, neat PLA pellets were masticated in a two-roll mill (191–TM, Yasuda Seiki Seisakusho Ltd., Tokyo, Japan) at approximately 160 ± 3 °C with a nip gap of 0.6 mm for 3 min. Subsequently, unmasticated or masticated NR was added, followed by mastication for an additional 3–5 min to produce the PLA/NR compounds. These were then remixed in an internal mixer at a set temperature of 130 °C, excluding the heat generated from the shear, with a rotor speed of 25–55 rpm for 15 min to obtain the PLA/NR blends. One should note that using a higher temperature may result in the rubber exhibiting a sticky texture, indicative of potential degradation. Finally, sheet samples of these blends were prepared via compression molding at 140 ± 5 °C for 10 min. The same process was applied in preparing the P-PLA/NR but with a slightly lower mixing temperature of around 150 ± 3 °C in the two-roll mill.

#### 2.4.2. Effect of P-PLA/NR Weight Ratio

The TPNR was prepared with varying PLA-to-NR weight ratios—60/40, 70/30, 80/20, and 90/10—to evaluate the influence of different rubber contents on the material properties. These blends were synthesized following the previously mentioned procedure. The NR was masticated for 60 min as this duration yielded the optimal mechanical properties.

### 2.5. Mechanical Properties

The tensile strength, % of elongation at break, Young’s modulus, and toughness were determined using a universal testing machine (Hounsfield, model H10KS, Redhill, UK). Dumbbell-shaped specimens with a thickness of 1 mm were prepared following the ASTM D412 [31] method and tested at a crosshead speed of 50 mm/min and a gauge length of 40 mm, using a 200 N load cell. The toughness was calculated from a stress–strain curve, obtained through a tensile test. The hardness was assessed using a Shore D durometer following ASTM D2240 (Frank GmbH, Mörfelden-Walldorf, Germany), applying a 50 N load for approximately 5 s. The impact strength (Izod impact test; IMP–25J, Chareontut Co., Ltd., Bangsaothong, Thailand) was evaluated according to ASTM D256 [18,30], using rectangular specimens prepared via compression molding, with a thickness of 2 mm, a width of 15 mm, a length of 75 mm, and a 45° notch 2 mm deep. The pendulum energy of 5.4 J was applied during the test.

### 2.6. Thermal Properties

The glass transition temperature (T_g_), cold crystallization temperature (T_cc_), T_m_, and degree of crystallinity (χ_c_) were examined using a differential scanning calorimeter (DSC; Mettler Toledo, Greifensee, Switzerland). The PLA and P-PLA compounds were studied over a temperature range of 25–200 °C, while the TPNR compounds were examined over a broader range of –80 to 200 °C. In both cases, the heating rate was set to 10 °C/min, with a nitrogen gas flow rate of 20 mL/min and a sample weight of 10–12 mg. The crystallinity of the samples was calculated according to Equation (1) [19]:

(1) $$\chi_{c}=\frac{{\Delta H}_{m}}{{\Delta H}_{m}^{0}\;\times \;\varphi_{\mathrm{PLA}}}\;\times 100,$$

where $\chi_{c}$ is the degree of crystallinity, ${\Delta H}_{m}$ is the enthalpy of fusion, ${\Delta H}_{m}^{0}$ is the enthalpy of fusion at a PLA crystallinity of 100% as 93 J/g [19], and $\varphi_{\mathrm{PLA}}$ is the weight fraction of PLA in the blends.

### 2.7. Morphological Properties

The fracture surfaces of the samples were examined using a scanning electron microscope (SU3900, Hitachi, Tokyo, Japan). In the preparation phase, the TPNR samples were first fractured in liquid nitrogen. The NR phase was then selectively removed by soaking the samples in toluene for 5 days [31]. After the extraction, the samples were dried in a hot-air oven, sputter-coated with a conductive material, and finally scanned at an accelerating voltage of 20 kV.

### 2.8. Dynamic Mechanical Properties

A dynamic mechanical thermal analyzer (DMTA; DMA 1, Mettler Toledo, Greifensee, Switzerland) was used to investigate the Tan δ of the materials. The analysis was performed in tension mode—with a constant heating rate of 3 °C/min, a strain amplitude of 10 µm, and a frequency of 1 Hz—over the temperature range of –100 to 120 °C [32].

### 2.9. M_w_ and Polydispersity Index

To prepare the samples for gel permeation chromatography (GPC), 0.01 g was dissolved in 10 mL of tetrahydrofuran (THF) and then filtered through a 0.45 µm membrane needle filter. The prepared solution was injected into a GPC system (1260GPC/SEC MDS, Agilent Technologies, Santa Clara, CA, USA) equipped with KF-806M and KF-803L columns. THF was used as the mobile phase at a flow rate of 1.0 mL/min. The analysis was conducted at 40 °C, using a refractive index detector for signal detection [33].

### 2.10. Rheological Measurements

The rheological properties of NR were measured according to ASTM D383 [11,34] using a capillary rheometer (RG25, Gottfert, Buchen, Germany) at a temperature of 160 °C and a shear rate ranging from 10 to 10,000 s^−1^. The rheological properties of NR were reported by plotting the shear viscosity (Pa.s) as a function of the shear rate (s^−1^).

### 2.11. Statistical Analysis

The data were analyzed using the Statistical Package for the Social Sciences (SPSS), version 22.0, and Duncan’s test was applied with a confidence level of *p* < 0.05.

## 3. Results and Discussion

### 3.1. Effect of ATBC and NR Mastication Time

#### 3.1.1. Morphological Properties

**Figure 1:**
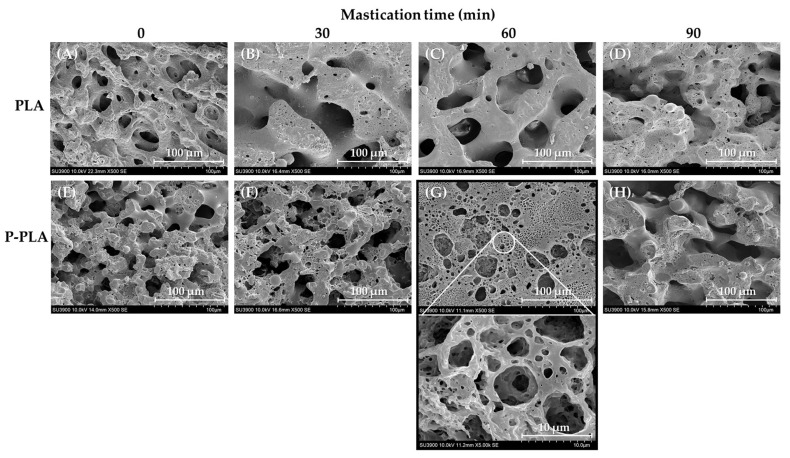
SEM micrographs of sample fracture surfaces, including (**A**–**D**) PLA/NR and (**E**–**H**) P-PLA/NR at a weight ratio of 60/40 with different mastication times: (**A**) PLA/NR, 0 min; (**B**) PLA/NR, 30 min; (**C**) PLA/NR, 60 min; (**D**) PLA/NR, 90 min; (**E**) P-PLA/NR, 0 min; (**F**) P-PLA/NR, 30 min; (**G**) P-PLA/NR, 60 min; and (**H**) P-PLA/NR, 90 min.

The scanning electron microscopy (SEM) micrographs in Figure 1 reveal co-continuous phases in both the PLA/NR and P-PLA/NR blends with varying NR mastication times. The PLA/NR blends (Figure 1A–D) exhibit large NR phase sizes within the PLA matrix, indicating significant immiscibility and phase separation between PLA and NR. This is primarily due to the substantial polarity differences between NR and PLA, further exacerbated by the high NR content [23]. In contrast, Figure 1E–G demonstrate a reduction in the NR phase size when using P-PLA as the polymer matrix. The P-PLA/NR blend with an NR mastication time of 60 min (Figure 1G) displays the smallest NR phase size. However, increasing the mastication time to 90 min (Figure 1H) results in a larger NR phase size compared to the other samples; this is attributed to the breakdown and subsequent agglomeration of recombined NR chains during the extended mastication [11,35].

#### 3.1.2. Mechanical Properties and M_w_

**Figure 2:**
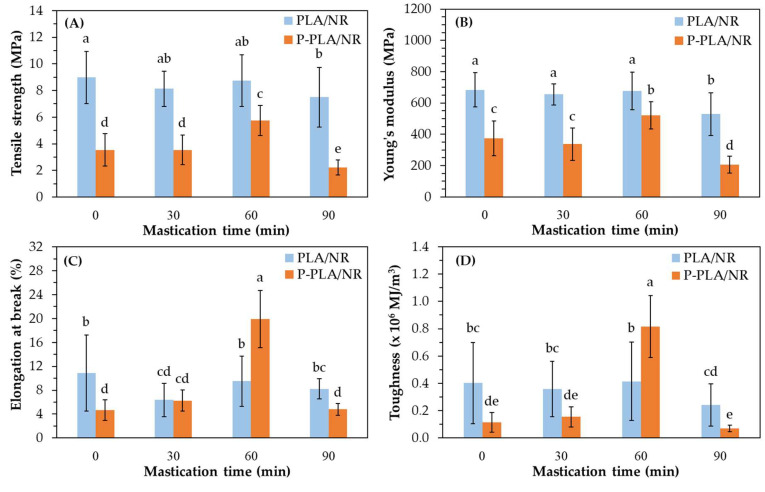
(**A**) Tensile strength, (**B**) Young’s modulus, (**C**) elongation at break, and (**D**) toughness of PLA/NR and P-PLA/NR (weight ratio of 60/40) at different NR mastication times. Different superscript letters (a–e) represent statistically significant differences (*p* ≤ 0.05) between the bars.

Figure 2 shows the tensile strength, elongation at break, Young’s modulus, and toughness of PLA/NR and P-PLA/NR for different NR mastication times (0, 30, 60, and 90 min) at a weight ratio of 60/40. Compared to the case of P-PLA/NR, the tensile strength and Young’s modulus of PLA/NR remain relatively high across all NR mastication times because PLA, which is not plasticized, typically exhibits high crystallinity and greater strength [3]. However, as the NR mastication time increases, the tensile strength of PLA/NR decreases; this is attributed to mechanical shear scission, leading to a general decrease in the M_w_ of the NR, as shown in Table 3. Similarly, masticating the NR does not result in an increase in the elongation at break and the toughness of the PLA/NR material. In comparing the experimental results with previous studies employing a lower NR content (1–10 wt%), the outcomes initially appear inconsistent [5,9,10,11]. However, the differences could be attributed to higher NR content leading to increased phase separation and significantly impairing the mechanical properties [23]. This effect outweighs any potential benefits from the reduction in the molecular weight, which might otherwise have enhanced compatibility. Bitinis et al. (2011) [9], Pongtanayut et al. (2013) [10], and Pattamaprom et al. (2016) [11] reported that the elongation at break and the toughness of materials increases as the NR contents increase from 3 to 10 wt%. However, they found that higher NR contents decrease both properties due to the formation of larger NR droplets. Additionally, Zhang et al. (2013) [5] also indicated that incorporating 1–3% NR into PLA can improve the elongation at break and the toughness of PLA.

Increasing the NR mastication time significantly influences the mechanical properties of P-PLA/NR. Extending this time to 60 min increases the tensile strength from approximately 3.54 to 5.75 N/mm^2^; it also increases Young’s modulus from 374.17 to 521.33 MPa and the toughness from 0.11 to 0.82 ×10^6^ MJ/m^3^, compared to the blend of unmasticated NR. The experimental results can be attributed to several factors. First, given its reduced crystallinity, P-PLA may have blended more effectively with NR, an amorphous polymer, at an optimal molecular weight of the NR. This is due to differences in crystallinity potentially leading to phase separation in polymer blends comprising incompatible polymers [36]. Additionally, ATBC, used as a PLA plasticizer, may have served as a compatibilizer in this polymer blend. The dual role of ATBC enhances the dispersion of NR within the P-PLA matrix, leading to a more homogeneous material. The improved compatibility and dispersion are corroborated by SEM images revealing enhanced interfacial interactions and a refined phase morphology, as illustrated in Figure 1G.

**Scheme 1:**
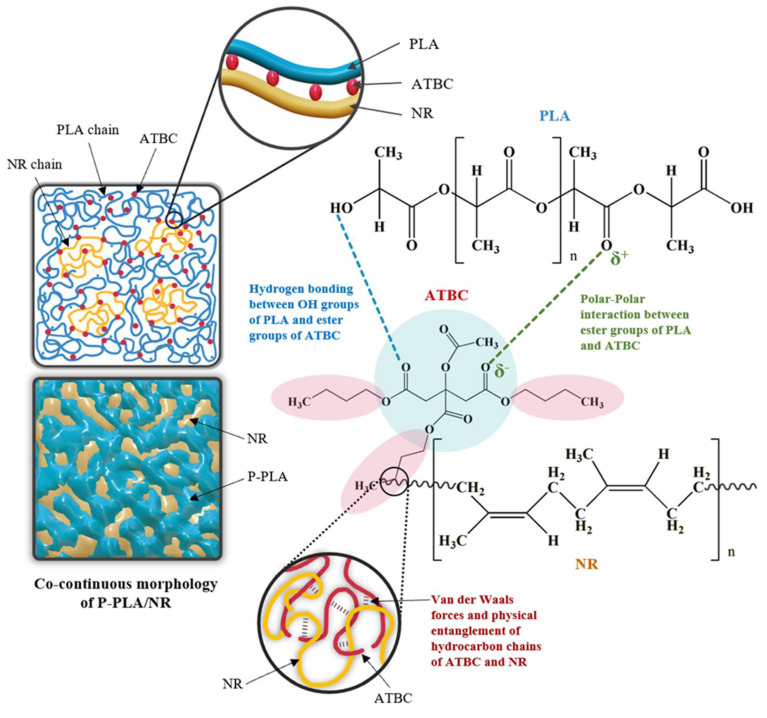
Proposed interaction mechanisms among PLA, ATBC, and NR, depicting the chemical and physical interactions.

To further elucidate the observed improvements in material homogeneity and compatibility, a model of the potential chemical interactions was proposed, as illustrated in Scheme 1. This model suggests that ATBC molecules interact with both PLA and NR, creating a compatible interface that promotes the formation of tiny droplets and a more uniform dispersion of NR distributed in the PLA matrix. Specifically, PLA and ATBC both contain ester functional groups, which play a significant role in their interactions. PLA contains ester linkages within its polymer backbone, while ATBC is an ester derivative of citric acid; their ester groups can engage in hydrogen bonding and other polar interactions [37], enhancing the compatibility between the two polymers. In contrast, the interaction between ATBC and NR is characterized by van der Waals forces and the physical entanglement of hydrocarbon chains. This functionality of ATBC helps form a network where ATBC molecules bridge the PLA and NR phases, thereby enhancing the mechanical properties of the TPNR blends. The greater compatibility and dispersion are confirmed by SEM images, revealing the presence of enhanced interfacial interactions and a refined phase morphology, particularly the co-continuous phase structure of the TPNR blends.

In comparison to conventional copolymers utilized for compatibilization, such as ethylene-methyl acrylate-glycidyl methacrylate (EMA-GMA) and polyolefin elastomer-grafted glycidyl methacrylate (POE-g-GMA), ATBC presents several advantages, including low toxicity, renewable origins, and biodegradability [15,21,22]. Traditional compatibilizers often rely on petrochemical-derived compounds and may necessitate additional processing steps, or exhibit limited biodegradability, which compromises their sustainability credentials. In contrast, ATBC, derived from renewable sources, aligns with the principles of green chemistry and circular economy practices. Furthermore, ATBC serves as a plasticizer, whereas copolymers primarily function to enhance compatibility. However, it is crucial to acknowledge that copolymers may exhibit superior compatibility-enhancing performance owing to their chemically modified structures, which are specifically modified to optimize blending with diverse polymer types.

However, after a mastication time of 90 min, the mechanical properties of NR (Figure 2A–D) significantly deteriorate, reaching their lowest values compared to the cases for shorter mastication periods (0–60 min). This decline occurs even though the M_w_ is only slightly lower than that of NR masticated for 60 min (Table 3), which could be explained by the prolonged mastication time increasing the exposure of the NR chains to oxygen. This leads to the formation of free radicals that attack the polymer backbone, causing chain cleavage rather than the application of shear forces—a phenomenon known as the thermo-oxidative effect [38,39]. In addition, oxidative degradation often results in the formation of polymer chains that cross-link or recombine into longer molecules with potentially weaker structures [40]. This effect results in the absence of significant differences in the M_w_ of the NR masticated for 60 and 90 min.

**Figure 3:**
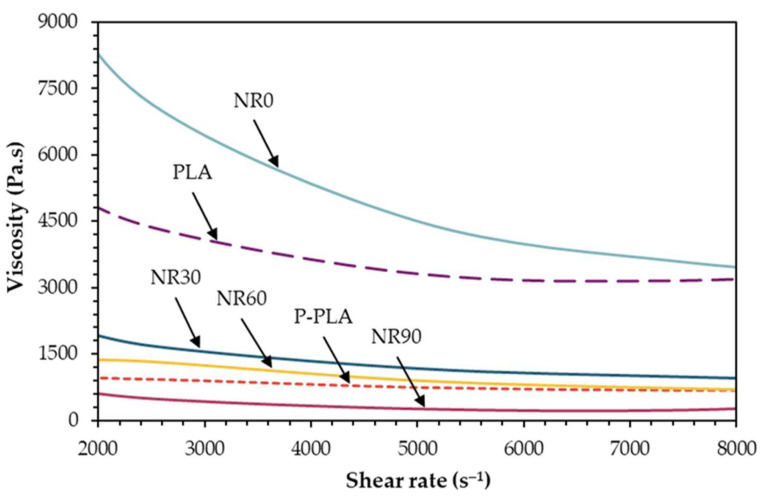
Relationship between viscosity and shear rate for PLA, P-PLA, unmasticated NR (0 min, NR0), and masticated NR (with mastication times of 30, 60, and 90 min, corresponding to NR30, NR60, and NR90, respectively).

To validate this explanation, shear viscosity measurements were performed on all NR samples using a capillary rheometer, as shear viscosity is a critical parameter in determining the processability, phase structure, and performance of polymer blends [41]. All masticated NR samples exhibit a lower shear viscosity than the unmasticated NR, which has a higher M_w_, as revealed by the observed trend. However, P-PLA exhibits a lower shear viscosity than PLA and is more comparable to that of NR masticated for 60 min, with the values further converging as the shear rate increases (Figure 3). This similarity in the shear viscosity could enhance the mixing efficiency during processing and reduce phase separation by enabling more effective stress transfer between phases [11]. An NR mastication time of 60 min was determined as the most suitable for blending with P-PLA. While extended mastication times do not significantly alter the molecular weight, the shear viscosity is lower than that of NR masticated for 60 min. This reduction in viscosity is attributed to prolonged oxygen exposure during mastication, which leads to shear-induced chain scission through oxidative degradation [40]. The fragmented polymer chains form unstable intermediates with diminished mechanical integrity; when these chains recombine, the resulting polymer exhibits a reduced shear viscosity and lower mechanical properties [42,43].

**Figure 4:**
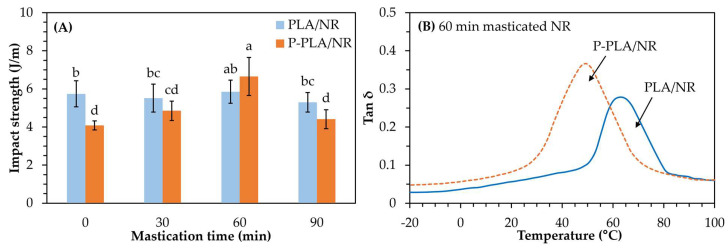
(**A**) Impact strength of PLA/NR and P-PLA/NR blends at a weight ratio of 60/40 with varying NR mastication times, and (**B**) Tan δ curves of blends using NR masticated for 60 min. Different superscript letters (a–d) in Figure 4A present statistically significant differences (*p* ≤ 0.05) between the bars.

In Figure 4A, the impact strength of the blends displays a trend similar to that of the toughness (shown in Figure 2D), with a maximum value of 6.65 J/m for an NR mastication time of 60 min. This is attributed to the improved compatibility of the material compared to other samples, as previously discussed. Additionally, the incorporation of ATBC enhances chain mobility, increases the viscous phase, and improves energy absorption [44]. This leads to higher damping properties, as reflected by the Tan δ values (Figure 4B), representing the ratio of the loss modulus (viscous component) to the storage modulus (elastic component). Experimental results exhibit a similar trend, with higher Tan δ values after the addition of 5.0 phr paraffin oil plasticizer to NR-based elastomers [45]. This trend also aligns with the findings of Dominguez-Candela et al. (2021) [46], who reported that adding a plasticizer (epoxidized chia seed oil, 2.5−10.0%) to PLA increases the intensity of the Tan δ peak due to the plasticizing effect.

### 3.2. Effect of P-PLA/NR Weight Ratio

#### 3.2.1. Morphological Properties

**Figure 5:**
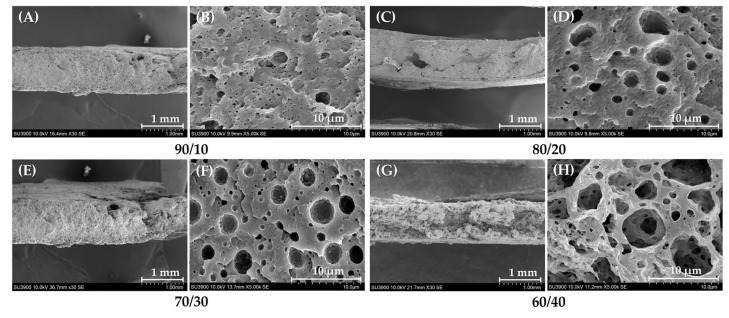
SEM micrographs of P-PLA/NR fracture surfaces at various P-PLA/NR weight ratios, displayed at low and high magnifications: (**A**,**B**) 90/10, (**C**,**D**) 80/20, (**E**,**F**) 70/30, and (**G**,**H**) 60/40.

Regarding the P-PLA/NR blend ratios, the NR that masticated for 60 min was selected to prepare the TPNR materials, as it yielded the highest mechanical properties, according to the results presented in Section 3.1. The P-PLA/NR ratio was varied as follows: 90/10, 80/20, 70/30, and 60/40. Figure 5 presents SEM micrographs of the dispersion of masticated NR within P-PLA. The low-magnification SEM images reveal rough and distinct interfaces between the NR and P-PLA phases with an increase in the NR content, indicative of poor miscibility. Conversely, at a lower NR content, they exhibit smoother and more integrated interfaces, as shown in Figure 5A,C,E,G. Furthermore, at a higher magnification, the SEM images depict the impact of different polymer weight ratios on the dispersion pattern (Figure 5B,D,F,H). An increase in the NR content leads to the formation of larger domains with coarser dispersion throughout the P-PLA matrix due to the polarity difference between PLA and NR [23]. On the other hand, as the NR content decreases, the NR phase exhibits a finer and more uniform dispersion, suggesting enhanced compatibility between the components [9,10,11].

#### 3.2.2. Mechanical Properties

Table 4 summarizes the mechanical properties of neat PLA and the P-PLA/NR blends with varying NR contents. Neat PLA exhibits the highest tensile strength and Young’s modulus, attributed to its high crystallinity and rigid structure [3,47]. However, introducing 15 wt% of the plasticizer (ATBC) into the PLA matrix (100/0 P-PLA/NR or P-PLA) leads to a notable reduction in the material strength. This decline can be explained by considering that the plasticizer disrupts the intermolecular forces between the polymer chains, leading to a decrease in both the tensile strength and Young’s modulus [48]. In contrast, a significant increase in the elongation at break was observed, reaching ~246.6%; this indicates enhanced flexibility due to the plasticizing effect of ATBC, which improves chain mobility. The toughness also rises sharply from 1.61 MJ/m^3^ in neat PLA to 44.19 MJ/m^3^, reflecting the improved ability of the material to absorb and dissipate energy under stress [49].

In the P-PLA/NR blends, the mechanical properties progressively decline as the NR content increases due to the soft and elastic nature of NR, which disrupts the rigidity of PLA [10]. Furthermore, the polymers are not fully compatible, resulting in phase separation into distinct regions, driven by composition and polarity differences. This phase separation weakens the mechanical performance, reducing the strength, elongation at break, and toughness of the material due to poor interfacial adhesion [23]. The hardness values also follow a downward trend with increasing NR content, reflecting the softer characteristics of NR. However, among all the polymer blends, the 90/10 P-PLA/NR one exhibits the highest overall mechanical properties, comparable to those reported by Bitinis et al. (2011), Pongtanayut et al. (2013), and Pattamaprom et al. (2016) [9,10,11]. This is attributed to the blend exhibiting the lowest degree of phase separation, as evidenced by SEM images (Figure 5A,B).

Table 5 presents notable trends in the impact strength, fracture percentage, and failure mode of P-PLA/NR blends with varying NR contents. Neat PLA displays the lowest impact strength and a fracture rate of 100%, indicating that it is highly brittle and absorbs minimal energy before completely fracturing. Incorporating a small amount of plasticizer (ATBC, 15 wt%) leads to a slight improvement in the impact strength, as plasticization typically shifts the material behavior from brittle to more ductile [50]. However, the fracture rate remains at 100%; this indicates that despite the improvements, the material remains brittle in terms of its maximum energy absorption under the impact force, as it does not undergo plastic deformation at the notch before fracturing.

**Figure 6:**
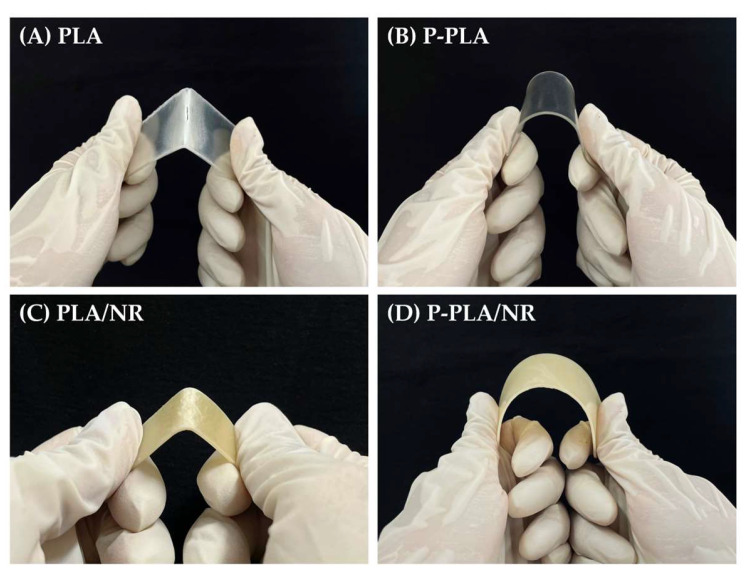
Images of sheet samples after bending: (**A**) neat PLA, (**B**) P-PLA, (**C**) PLA/NR blend, and (**D**) P-PLA/NR blend, with both (**C**,**D**) involving a weight ratio of 90/10.

Conversely, incorporating NR into P-PLA significantly enhances its toughness, even at low concentrations (10−20 wt%). Specifically, the addition of 10% NR significantly increases the impact strength, making it approximately 6−8-fold greater than that of neat PLA and P-PLA. The fracture percentage decreases substantially, reaching 6.77%, with no breakage failure, indicating a notable improvement in toughness and flexibility due to the high energy absorption capacity of NR [51]. These findings align with previous research by Pattamaprom et al. (2016) [11]. Figure 6 further confirms that the 90/10 P-PLA/NR blend exhibits superior flexibility and can withstand significant deformation without cracking under applied stress, in contrast to the neat PLA and the unplasticized PLA/NR blend.

The impact strength of the 80/20 P-PLA/NR blend is lower than that of the 90/10 P-PLA/NR blend but still significantly higher than that of neat PLA and P-PLA. Beyond this concentration, the toughness declines significantly, and the fracture rate returns to 100%. This suggests that a high NR content leads to poor interfacial bonding between the NR and PLA, resulting in reduced compatibility and the diminished ability of the TPNR to distribute stress and maintain toughness [9].

#### 3.2.3. Thermal Properties

**Figure 7:**
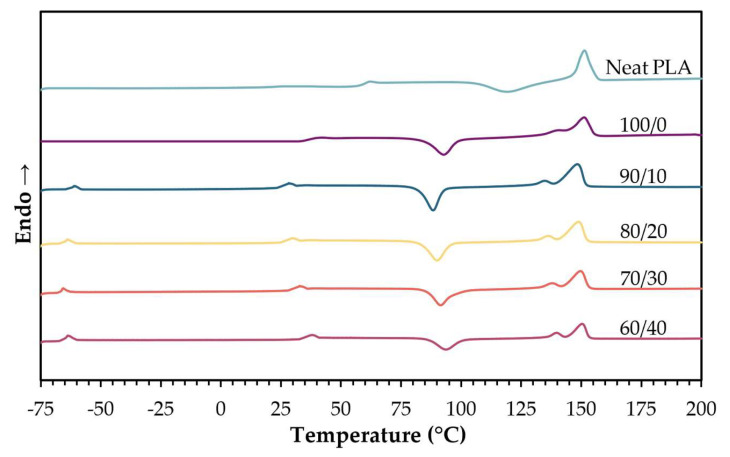
DSC curves of neat PLA, P-PLA, and P-PLA/NR at different weight ratios.

Figure 7 and Table 6 present the results from the first DSC heating scan for neat PLA, P-PLA, and P-PLA/NR blends with varying weight ratios. P-PLA exhibits lower T_g_ and T_m_ values and a reduced degree of crystallinity compared to neat PLA, indicating that P-PLA becomes more flexible at room temperature. This behavior is attributed to the addition of plasticizers, which increase the free volume within the polymer matrix and maintain it upon cooling [52,53,54].

The P-PLA/NR blends all exhibit a lower T_g_ compared to P-PLA. This reduction is due to the incorporation of NR chains, creating additional free space for the PLA chains and, thus, facilitating their movement [5]. The blends containing 10 to 40 wt% NR show two distinct T_g_ peaks, corresponding to NR and P-PLA; this indicates the immiscibility of the two components and the formation of separate phases [55]. Additionally, the T_g_ values of the polymers shift further apart with increases in the NR content, which might indicate a tendency toward increased phase separation within the polymer blends [56].

The degree of crystallinity in P-PLA decreases from approximately 31.82 to 25.16% as the NR content increases from 10 to 40 wt%. This reduction is attributed to the increased amorphous nature of NR, enhanced chain mobility, and the phase separation between P-PLA and NR, particularly at higher NR levels [57,58]. Additionally, the T_cc_ of the P-PLA/NR blends increases at higher NR contents, suggesting that greater levels of energy and higher temperatures are required for crystallization due to increased chain mobility [58].

In comparison to the work of Burkov et al. (2021) [13], who used epoxidized soybean oil (15 wt%) as both a plasticizer and compatibilizer in PLA/NR polymer blends (60/40), the observed difference between epoxidized soybean oil and ATBC is that the former appears to enhance material compatibility more effectively through its epoxy groups. This is evident from the higher tensile strength (9.8 MPa) and elongation at break (52%) at the same blend ratio. In contrast, ATBC is more effective in reducing the thermal properties of the materials, suggesting that ATBC may perform better as a plasticizer. The T_g_ and T_m_ values of P-PLA/NR using ATBC are 32.83 °C for the PLA matrix and 135.06 °C, respectively. In contrast, using epoxidized soybean oil results in a T_g_ for the PLA phase that is approximately 18 °C higher, along with an increase in T_m_ by 13 °C compared to ATBC.

## 4. Conclusions

This study demonstrated that the NR mastication time, the use of a plasticizer (ATBC), and the weight ratio significantly affect the properties of PLA-based TPNR blends. The optimal NR mastication time for preparing the P-PLA/NR blends was 60 min, and extending mastication beyond this period resulted in increased phase separation. The NR mastication time did not affect the mechanical properties of the unplasticized PLA/NR blends, and a high NR content led to greater phase separation. The addition of the plasticizer ATBC (15 wt%) to PLA before TPNR preparation can lead to improved compatibility and enhanced mechanical properties, such as higher ductility and impact resistance, with ATBC acting as both a plasticizer and a compatibilizer. Therefore, selecting an appropriate plasticizer was crucial for developing flexible polymer blends. The P-PLA/NR blend at a ratio of 90/10 exhibited the highest elongation at break (65.06%) and impact resistance (35.40 J/m), approximately 6–8-fold higher than that of PLA and other P-PLA blends. This makes it suitable for applications requiring high impact resistance. Additionally, the incorporation of NR into P-PLA reduced the brittleness and T_g_ of the materials, increasing their suitability for use in low-temperature environments. Given that the material is derived from biodegradable polymers and is free from hazardous chemicals, it offers significant potential as eco-friendly food packaging. However, a limitation of this work is its applicability in high-temperature environments or situations requiring high durability, as the material has not undergone the vulcanization process. Additionally, long-term stability and environmental factors were not considered in this study. Future research will address these to evaluate the materials’ performance in real-world conditions and explore their potential in various environments.

## Figures and Tables

**Table 1:** Mechanical properties of P-PLA with different ATBC contents.

Property	Plasticizer Contents (wt%)			
	0	5	10	15
Tensile strength (MPa)	57.50 ± 3.10 ^a^	53.02 ± 4.69 ^b^	49.08 ± 3.01 ^c^	25.96 ± 3.17 ^d^
Elongation at break (%)	6.95 ± 0.52 ^b^	7.11 ± 0.70 ^b^	6.81 ± 0.61 ^b^	234.44 ± 12.84 ^a^
Young’s modulus (MPa)	1441 ± 101 ^a^	1239 ± 134 ^b^	1235 ± 102 ^b^	954 ± 132 ^c^
Toughness (10^6^ MJ/m^3^)	2.52 ± 0.33 ^b^	2.24 ± 0.27 ^b^	2.08 ± 0.27 ^b^	38.54 ± 6.08 ^a^
Hardness (Shore D)	81.00 ± 1.73 ^a^	78.60 ± 0.55 ^b^	75.60 ± 0.55 ^c^	70.60 ± 2.41 ^d^
Impact strength (J/m)	4.94 ± 0.82 ^a^	3.88 ± 0.84 ^b^	4.15 ± 0.47 ^b^	5.12 ± 0.75 ^a^

Different superscript letters (a–d) within a row represent statistically significant differences (*p* ≤ 0.05). The tensile properties, hardness, and impact strength (Izod impact test) were measured according to ASTM D882 [28], ASTM D2240 [29], and ASTM D256 [18,30], respectively.

**Table 2:** Experimental conditions.

Runs	Factor A (ATBC Contents, wt%)	Factor B (Mastication Time, min)
1	0 (Neat PLA)	0
2		30
3		60
4		90
5	15 (P-PLA)	0
6		30
7		60
8		90

**Table 3:** M_w_ and polydispersity index (PDI) of NR subjected to different mastication times, neat PLA, and P-PLA containing 15 wt% ATBC.

Sample	Mastication Time (min)	M_w_ (g/mol)	PDI
Unmasticated NR	0	1,402,766	5.29
Masticated NR	30	324,491	2.25
	60	296,152	2.02
	90	289,397	2.16
Neat PLA		150,181	1.71
P-PLA		156,344	1.90

**Table 4:** Mechanical properties of neat PLA and P-PLA/NR at different weight ratios.

Sample	Tensile Strength (MPa)	Young’s Modulus (MPa)	Elongation at Break (%)	Toughness (×10^6^ MJ/ m^3^)	Hardness (Shore D)
Neat PLA	41.23 ± 3.50 ^a^	1914 ± 19 ^a^	6.85 ± 0.66 ^e^	1.61 ± 0.18 ^d^	81.00 ± 1.73 ^a^
P-PLA/NR					
100/0 *	39.35 ± 2.94 ^b^	787 ± 94 ^b^	246.58 ± 10.90 ^a^	44.19 ± 4.16 ^a^	70.60 ± 2.41 ^b^
90/10	16.74 ± 0.80 ^c^	668 ± 72 ^c^	65.06 ± 32.18 ^b^	7.21 ± 3.58 ^b^	65.20 ± 0.45 ^c^
80/20	13.37 ± 0.96 ^d^	643 ± 47 ^c^	46.54 ± 23.54 ^c^	4.21 ± 1.83 ^c^	59.20 ± 0.84 ^d^
70/30	9.10 ± 1.13 ^e^	517 ± 73 ^d^	23.56 ± 12.09 ^d^	1.52 ± 0.83 ^d^	53.60 ± 0.89 ^e^
60/40	5.75 ± 1.13 ^f^	521 ± 86 ^d^	19.93 ± 4.80 ^de^	0.82 ± 0.23 ^d^	48.00 ± 0.71 ^f^

* The 100/0 P-PLA/NR blend denotes P-PLA. Different superscript letters (a–f) within a column represent statistically significant differences (*p* ≤ 0.05).

**Table 5:** Impact strength of neat PLA and P-PLA/NR at different weight ratios.

Sample	Impact Strength (J/m)	% Fracture	Type of Failure	
Neat PLA	4.19 ± 0.25 ^e^	100 ± 0 ^a^	C	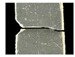
P-PLA/NR				
100/0 *	5.85 ± 0.57 ^c^	100 ± 0 ^a^	C	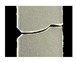
90/10	35.40 ± 2.56 ^a^	6.77 ± 0.98 ^c^	NB	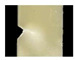
80/20	15.37 ± 3.41 ^b^	46.92 ± 9.03 ^b^	NB	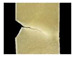
70/30	5.40 ± 0.56 ^cd^	100 ± 0 ^a^	C	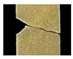
60/40	5.18 ± 0.22 ^d^	100 ± 0 ^a^	C	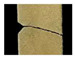

* The 100/0 P-PLA/NR blend denotes pure P-PLA. Different superscript letters (a–e) within a column represent statistically significant differences (*p* ≤ 0.05). The type of failure for each sample is specified according to ASTM D256 [30]: complete break (C)—specimen broken into two separate pieces; non-break (NB)—specimen exhibits a fracture of less than 90%, as measured from the vertex of the notch to the opposite side of the specimen.

**Table 6:** Thermal properties of neat PLA, P-PLA, and P-PLA/NR at different weight ratios.

Sample	T_g_ (°C)		T_m_ (°C)	T_cc_ (°C)	$\chi_{c}$ (%)
	NR	PLA			
Neat PLA	**-**	56.43	150.39	106.40	42.25
P-PLA/NR					
100/0 *	-	33.86	134.14	84.59	31.82
90/10	–64.03	23.52	130.10	82.44	31.10
80/20	–67.17	24.95	131.14	83.38	29.97
70/30	–67.78	28.11	132.71	85.65	30.14
60/40	–66.93	32.83	135.06	86.45	25.16

* The 100/0 P-PLA/NR blend signifies pure P-PLA.

## Data Availability

All data of this study are included in this manuscript. Further inquiries can be directed to the corresponding authors.
